# *DLEC1* and *MLH1* promoter methylation are associated with poor prognosis in non-small cell lung carcinoma

**DOI:** 10.1038/sj.bjc.6604452

**Published:** 2008-07-01

**Authors:** T J Seng, N Currey, W A Cooper, C-S Lee, C Chan, L Horvath, R L Sutherland, C Kennedy, B McCaughan, M R J Kohonen-Corish

**Affiliations:** 1 Cancer Research Program, Garvan Institute of Medical Research, Sydney 2010, Australia; 2 Department of Anatomical Pathology, Royal Prince Alfred Hospital, Sydney 2050, Australia; 3 Discipline of Pathology, Central Clinical School and Bosch Institute, University of Sydney, Sydney 2006, Australia; 4 Department of Anatomical Pathology, Concord Hospital, Sydney 2139, Australia; 5 Sydney Cancer Centre, Royal Prince Alfred Hospital, Sydney 2050, Australia; 6 St Vincent's Clinical School, Faculty of Medicine, University of NSW, Sydney 2010, Australia; 7 Strathfield Private Hospital, Sydney 2135, Australia; 8 Department of Cardiothoracic Surgery, Royal Prince Alfred Hospital, Sydney 2050, Australia

**Keywords:** NSCLC, mismatch repair, DLEC1, promoter methylation, prognosis

## Abstract

The significance of chromosome 3p gene alterations in lung cancer is poorly understood. This study set out to investigate promoter methylation in the *deleted in lung and oesophageal cancer 1* (*DLEC1*), *MLH1* and other 3p genes in 239 non-small cell lung carcinomas (NSCLC). *DLEC1* was methylated in 38.7%, *MLH1* in 35.7%, *RARβ* in 51.7%, *RASSF1A* in 32.4% and *BLU* in 35.3% of tumours. Any two of the gene alterations were associated with each other except *RARβ*. *DLEC1* methylation was an independent marker of poor survival in the whole cohort (*P*=0.025) and in squamous cell carcinoma (*P*=0.041). *MLH1* methylation was also prognostic, particularly in large cell cancer (*P*=0.006). Concordant methylation of *DLEC1*/*MLH1* was the strongest independent indicator of poor prognosis in the whole cohort (*P*=0.009). However, microsatellite instability and loss of MLH1 expression was rare, suggesting that *MLH1* promoter methylation does not usually lead to gene silencing in lung cancer. This is the first study describing the prognostic value of *DLEC1* and *MLH1* methylation in NSCLC. The concordant methylation is possibly a consequence of a long-range epigenetic effect in this region of chromosome 3p, which has recently been described in other cancers.

Lung cancer is one of the most common causes of cancer death. The overall 5-year survival rate for surgical resection of stage I non-small cell lung cancer (NSCLC) can achieve 60–75% while survival rates in stage II–IV patients remain poor. Unfortunately only a small subset responds to currently available treatments. Thus, it is important to identify and characterise new molecular markers and gene targets to improve the accuracy of prognosis and develop more targeted treatment strategies to improve the clinical management of lung cancer.

Allelic loss of chromosome 3p is one of the most frequent and earliest documented events in lung cancer, with deletions at 3p24–26, 3p21.3, 3p21.1–21.2, 3p14.2 and 3p12–13, suggesting the presence of multiple tumour suppressor genes on 3p (Hung *et al*, 1995; Wistuba *et al*, 2000; Zabarovsky *et al*, 2002). Recent work has revealed the involvement of frequent epigenetic alterations in the inactivation of many 3p candidate genes, including *BLU*, *FHIT, RASSF1A*, *RARβ* and *SEMA3B* (Dammann *et al*, 2000; Virmani *et al*, 2000; Zochbauer-Muller *et al*, 2001; Zabarovsky *et al*, 2002; Ito *et al*, 2005). Detection of methylated genes in serum and sputum DNA from lung cancer patients has also raised the possibility of using DNA methylation as an early detection marker (Esteller *et al*, 1999; Palmisano *et al*, 2000; Belinsky *et al*, 2002; Usadel *et al*, 2002).

Methylation of the *MLH1* gene in 3p22.3 and its correlation with a mismatch repair defect and high microsatellite instability (MSI-H) is well characterised in sporadic colorectal cancer, where this phenotype is associated with better patient survival (Sinicrope *et al*, 2006). In NSCLC *MLH1* methylation has been described with frequencies ranging from 7 to 59% (Yanagawa *et al*, 2003; Safar *et al*, 2005) but in the absence of MSI-H (Benachenhou *et al*, 1998; Okuda *et al*, 2005). LOH within the *MLH1* gene has also been detected in 55% (Benachenhou *et al*, 1998) and reduced MLH1 expression in 59% of lung cancers (Xinarianos *et al*, 2000). These intriguing findings have been followed by a recent report that *MSH2*, but not *MLH1*, methylation is a marker of poor prognosis in a Taiwanese cohort of nonsmoking female NSCLC patients (Hsu *et al*, 2005). It remains to be determined if a mismatch repair gene defect has a role in lung carcinogenesis and why it is not associated with typical MSI-H.

The *deleted in lung and oesophageal cancer 1* (*DLEC1*) gene is located about 1 Mb centromeric from *MLH1* (Figure 1A). The 3p21.3 region was identified as one of the common deleted regions in lung cancer. Four candidate genes in this region were analysed but no evidence of their involvement in cancer development was found (Ishikawa *et al*, 1997). Further analysis led to the identification of the *DLC1* gene (Daigo *et al*, 1999), which was later renamed *DLEC1*. Loss of *DLEC1* expression has been observed in lung, oesophageal, renal, ovarian and nasopharyngeal carcinoma cell lines and primary tumours and functional analyses strongly suggest that *DLEC1* is a tumour suppressor gene (Daigo *et al*, 1999; Kwong *et al*, 2006, 2007). Promoter hypermethylation has been shown to be responsible for silencing of *DLEC1* in ovarian cancer and in nasopharyngeal carcinoma (Kwong *et al*, 2006, 2007) but there has been no comprehensive methylation analysis reported for lung cancer.

In this study, we investigated if promoter hypermethylation of *DLEC1* is found in lung cancer and whether it has any prognostic significance. We determined the relationship of *DLEC1* methylation with patient clinicopathologic variables and other 3p molecular markers, in particular *MLH1, RARβ*, *RASSF1* and *BLU* methylation.

## Patients and methods

### Lung cancer patients

We reviewed the NSCLC surgery database maintained by the one cardiothoracic surgeon (BMC) for the period of 1994–2000. Patients who had received induction chemotherapy or for whom sufficient tissue was not available, were excluded. The final cohort had 155 (64.9%) men and 84 women (35.1%) with a median age at diagnosis of 68 years (range, 41–87 years) and a median survival time of 36.9 months (range, 1–113 months). Data on survival was obtained from the Cancer Registry of NSW, by routine follow-up visits or contact with the patient's general practitioner. Overall survival was measured from the date of surgery to the date of death or the date of last follow-up, censored patients being those who were alive at the time of last follow-up.

This study cohort consisted of 92 (38.7%) adenocarcinomas (ADC), 54 (22.7%) large cell carcinomas (LCC), and 92 (38.7%) squamous cell carcinomas (SCC). These tumours were classified according to the American Joint Committee on Cancer (AJCC) tumour-node metastasis classification (Grondin and Liptay, 2002) and consisted of 153 (64.0%) stage I and 86 (36.0%) stage II tumours (Table 2). The study was approved by the Ethics Review Committee of the Royal Prince Alfred Hospital (approval no. X02-0216).

### DNA extraction and bisulphite treatment

Hematoxylin and Eosin-stained sections from paraffin-embedded tissue blocks were reviewed by an anatomical pathologist (WAC) for tumour and matching normal tissue specimens. Six to twelve serial 4 *μ*m sections of each block were used for DNA extraction, depending on the size of the tissue. DNA extraction was carried out using the Puregen Genomic DNA purification kit (Gentra Systems, MN, USA). Sodium bisulphite conversion was performed as previously described (Millar *et al*, 2002).

### Expression of DLEC1 in lung cancer cell lines

Five lung cancer cell lines, A427, A549, NCI-H292, NCI-H1299 and NCI-H358, were used. Total RNA and DNA were extracted from cell pellets using RNeasy® Mini Kit and DNeasy® Tissue Kit (Qiagen GmbH Inc., Germany), respectively. Normal human adult lung RNA samples were purchased from Stratagene (Stratagene, CA, USA). One microgram of RNA from each sample was used in a reverse transcription reaction using GeneAmp RNA PCR kit (Applied Biosystems, CA, USA). Expression of *DLEC1* was assessed by RT–PCR (DLEC1-F: 5′-TTCCTCCCTCGCCTACTC-3′; DLEC1-R: 5′-AAACTCATCCAGCCGCTG-3′). The primer pair was designed across exons 1 and 2 of the main *DLEC1* transcript NM_005106. *GAPDH* was used as control.

To investigate if methylation regulates expression of *DLEC1*, cancer cells were treated with 5-aza-2′-deoxycytidine, a DNA methyltransferase inhibitor. Freshly seeded cells were grown overnight in normal medium, which was then replaced with medium containing 1 *μ*M of 5-aza (Sigma-Aldrich Corporation, MO, USA). Cells were allowed to grow for 72 h, with 5-aza-containing medium changed every 24 h, and harvested for DNA and RNA extraction. A cell viability of >70% was retained after 72 h of treatment.

### Methylation-specific PCR

*Deleted in lung and oesophageal cancer 1* methylation status was assessed by a fluorescence based real-time detection quantitative methylation-specific PCR (MSP) with primers DLEC-m1, DLEC-m2 (Table 1) and a TaqMan® probe 5′-6FAM-TAATCAAACTTACGCTCACTTCGTCGCCG-BHQ1-3′ (Biosearch Technology, CA, USA) (Weisenberger *et al*, 2006). A reference gene *MYOD1* was employed to normalise the DNA input of each sample as previously described (Eads *et al*, 1999; Kohonen-Corish *et al*, 2007). Quantitative real-time PCR was performed for *DLEC1* and *MYOD1* in parallel using the RealMasterMix Probe ROX (Eppendorf, Hamburg, Germany) in the ABI7900HT Sequence Detection System (Applied Biosystems, CA, USA). *Deleted in lung and oesophageal cancer 1* methylation was scored as present when the value of (*DLEC1*/*MYOD1* × 100%)⩾5 or absent if the value is <5. All samples were run in duplicate.

Methylation-specific PCR of other chromosome 3p genes *RARβ*, *MLH1*, *RASSF1A* and *BLU* was carried out (Table 1) together with *MYOD1* amplification, as previously described (Eads *et al*, 1999; Kohonen-Corish *et al*, 2007). PCR steps included 30 s for denaturing, annealing and extension (40 cycles), initial denaturation and final elongation for 10 min, and annealing temperatures of 55°C (*MLH1*), 57°C (*MYOD1*), 63°C (*BLU*), and 58°C (*RARβ*, *RASSF1A*).

### Immunohistochemistry and MSI analysis

MLH1 expression on tissue microarrays was analysed as part of a previous study (Cooper *et al*, 2008). Matched normal bronchial mucosa or peripheral lung parenchyma specimens were used as control tissue for each patient. MLH1 expression was scored semiquantitatively by multiplying the percentage of cells showing nuclear expression and the intensity of staining using a 3-tier grading system (1=weak, 2=moderate and 3=strong staining). Reduced MLH1 expression was taken for a score less than 100, of the maximum score of 300. MSI was analysed as previously described (Kohonen-Corish *et al*, 2005, 2006), except that only two markers BAT25 and BAT26 were evaluated, which are sufficient for detecting high MSI (Suraweera *et al*, 2002).

### Statistical and survival analysis

Correlation between *DLEC1* methylation and clinicopathologic parameters was determined using the *χ*^2^ test while survival analysis was performed using the Kaplan–Meier log-rank and Cox Proportional Hazards Model in the StatView package, and *P*<0.05 was regarded as statistically significant. Only those variables that were significant predictors of survival outcome in univariate analysis were incorporated into multivariate analyses.

## Results

### High correlation between promoter methylation and loss of expression of DLEC1

**Figure 1:**
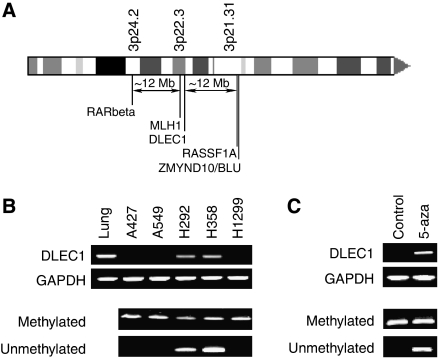
(**A**) Schematic drawing of the short arm of chromosome 3 and the relative location of the *RARβ*, *MLH1*, *DLEC1*, *RASSF1A* and *BLU* genes. (**B**) *DLEC1* (NM_005106) and *GAPDH* expression using RT–PCR (two upper panels) and methylation status using MSP (two bottom panels) in lung cancer cell lines and in normal human lung tissue. (**C**) Restoration of *DLEC1* expression and concomitant demethylation of the CpG island in H1299 cells using the 5-aza treatment.

Expression of *DLEC1* was assessed by RT–PCR in five lung cancer cell lines. While *DLEC1* was expressed in normal lung tissue, no expression was detected in the A427, A549 and H1299 lung cancer cell lines (Figure 1B). We assessed *DLEC1* methylation using methylation-specific PCR (MSP). Only the methylated allele was detected in the three cell lines where *DLEC1* was not expressed, while both the unmethylated and methylated alleles were detected in cell lines expressing *DLEC1* (Figure 1B). Methylation was rare in normal lung tissue (2.5%, 200 specimens analysed). To determine whether methylation directly regulates the silencing of *DLEC1*, the cell line H1299 was treated with 5-aza, a DNA methyltransferase inhibitor. After 3 days of 5-aza treatment, *DLEC1* expression was restored and demethylation observed (Figure 1C).

### Promoter methylation of DLEC1, MLH1, RAR*β*, RASSF1A and BLU in lung cancer

We employed MSP to assess the promoter methylation status of the five 3p candidate genes in 239 NSCLCs. Methylation was detected in 123 patients (51.5%) for *RARβ*, 86 (36.0%) for *MLH1*, 93 (38.9%) for *DLEC1*, 78 (32.6%) for *RASSF1A* and 85 (35.6%) for *BLU* (Table 2). Next we investigated the relationship between methylation of each set of two out of five genes. Significant correlation was observed between *DLEC1* and *MLH1* (*P*=0.0002), *DLEC1* and *RASSF1A* (*P*=0.0003), and *RASSF1A* and *BLU* methylation (*P*=0.017). *MLH1* methylation was also associated with *RASSF1A* (*P*=0.0006) and *BLU* (*P*=0.0005) (Table 3). Methylation of at least one of the five genes was detected in 204 of 239 (85.4%) patients; methylation of at least two genes in 139 (58.2%); three genes in 77 (32.2%); four genes in 36 (15.1%); and methylation of all five genes was detected in only nine (3.8%) patients.

### MLH1 expression in lung cancer tissue and MSI

Expression of *MLH1* was previously determined using immunohistochemistry on tissue microarrays in 105 of the 239 patients (Cooper *et al*, 2008). MSI was analysed in the whole cohort of 239 patients. Reduced *MLH1* expression was detected in seven of the 105 cancers including an apparent loss of MLH1 expression in two cancers, but none of the matching DNA specimens prepared from a larger area of the tumour showed any MSI using markers BAT25 and BAT26. Also, none of the seven cancers with reduced MLH1 expression showed *MLH1* promoter methylation. In the rest of the cohort MSI-H was detected in a stage 1B ADC (one marker) and a stage 2A LCC (both markers), of which only the latter was methylated in *MLH1*. There was no significant correlation between reduced MLH1 expression and survival (*P*=0.421).

### Methylation of DLEC1 and MLH1 are associated with poor patient survival

A statistically significant association between methylation and histologic type was observed, where *MLH1* methylation had a higher frequency in SCC (45.6%) and LCC (40.7%) compared with ADC (22.8%); *RASSF1A* methylation was associated with LCC (53.7%); *BLU* and *RARβ* methylation with ADC (45.7% and 60.9%). Furthermore, *MLH1* and *DLEC1* methylation were associated with the presence of regional lymph-node metastases and AJCC stage II. No association was observed between methylation of the five genes and age of diagnosis, gender or tumour differentiation status, except that *BLU* methylation was more common in older patients (Table 2).

**Figure 2:**
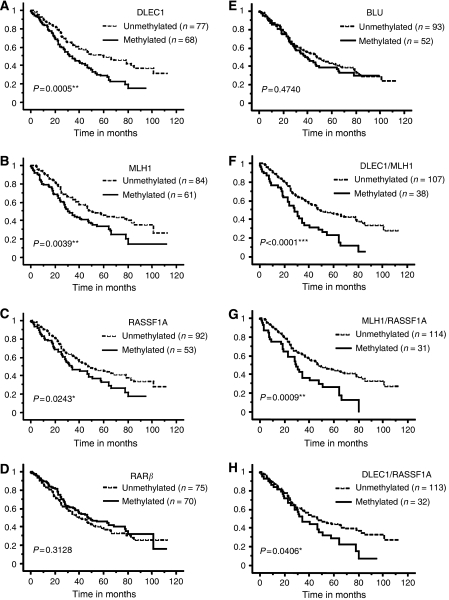
Kaplan–Meier log-rank analysis of overall survival of NSCLC patients stratified by promoter methylation of (**A**) *DLEC1*; (**B**) *MLH1*, (**C**) *RASSF1A*, (**D**) *RARβ*, (**E**) *BLU*, (**F**) *DLEC1/MLH1*, (**G**) *MLH1/RASSF1A* and (**H**) *DLEC1/RASSF1A* (^*^P<0.05; ^**^P<0.005; ^***^P<0.0001).

Methylation of *DLEC1* (*P*=0.0005), *MLH1*, (*P*=0.004), and *RASSF1A* (*P*=0.024) as well as regional lymph node status (*P*<0.0001) and AJCC stage (*P*<0.0001) were associated with poorer overall survival (Figure 2 and Table 2). *RARβ* and *BLU* methylation were not prognostic in the whole NSCLC cohort using the Kaplan–Meier log-rank analysis (*P*=0.313 and 0.474). Regional lymph node metastases and AJCC stage are two of the known prognostic factors for NSCLC and these two parameters are dependent predictors of survival in our cohort. Therefore, a bivariate analysis with the molecular marker predictor (*DLEC1*, *MLH1* or *RASSF1A* methylation) and AJCC stage was set up. Methylation of either *DLEC1* or *MLH1* but not *RASSF1A* was a prognostic indicator independent of AJCC stage in the entire patient cohort (Table 4). *Deleted in lung and oesophageal cancer 1* methylation was also a prognostic factor independent of AJCC stage in the SCC subgroup of patients (HR, 1.754; 95% CI, 1.023–3.007; *P*=0.041) and *MLH1* methylation in LCC (HR, 2.926; 95% CI, 1.358–6.308; *P*=0.006).

We then investigated if concordant methylation of two genes affect patient prognosis (Figure 2; Table 4). Concordant *MLH1*/*DLEC1* methylation was associated with poorer overall survival in both univariate (HR, 2.075; 95% CI, 1.428–3.015; *P*=0.0001) and bivariate (HR, 1.668; 95% CI, 1.138–2.447; *P*=0.009) analyses. Also, *MLH1* methylation was prognostic in combination with *RASSF1A* methylation independent of AJCC stage in all patients (HR, 1.688, 95% CI, 1.127–2.529; *P*=0.011) and particularly in the LCC cohort (HR, 3.223; 95% CI, 1.482–7.008; *P*=0.003).

## Discussion

*Deleted in lung and oesophageal cancer 1* is a candidate tumour suppressor gene in multiple cancers. Although the function of *DLEC1* is unclear, it suppresses tumour growth or reduces invasiveness of cancer cells (Daigo *et al*, 1999; Kwong *et al*, 2006, 2007). In this study, we demonstrate for the first time that the *DLEC1* promoter is methylated in lung cancer. The demethylating agent 5-aza reversed loss of mRNA expression in lung cancer cell lines. Frequent *DLEC1* methylation (34.2%) was observed in NSCLC and was most common in SCC (47.8%). *DLEC1* methylation was cancer-specific, as it was only rarely detected in matching normal lung tissue, and was strongly associated with stage II tumours and the spread of cancer to regional lymph nodes (*P*<0.0001). *DLEC1* methylation was also associated with shorter overall survival in the whole cohort and in the SCC group of patients, and this remained statistically significant upon bivariate analysis with AJCC stage (Table 4). As there is no antibody available for *DLEC1*, we could not determine what proportion of methylated tumours would show loss or reduced DLEC1 protein expression. However, it has been previously demonstrated that *DLEC1* RNA expression was lost in eight of 30 primary lung cancers and that this was not due to gene mutations (Daigo *et al*, 1999).

The *MLH1* gene is located within 1 Mb of *DLEC1* in a locus that shows 55% LOH in NSCLC (Benachenhou *et al*, 1998). Therefore, there has been some interest in determining the biological significance of reduced *MLH1* gene expression and promoter methylation in lung cancer. As gene alterations can cause either increased sensitivity or resistance of tumours to chemotherapy treatment, we excluded those patients who had received induction chemotherapy prior to surgery to avoid a possible bias in the molecular analyses. *MLH1* methylation was found in 36% of the cancers but did not result in the loss of gene expression in the 105 cancers analysed with immunohistochemistry. Only 6.7% of the cancers showed reduced MLH1 expression with stringent criteria (<100 of the maximum score of 300) and none of these specimens were methylated. We also found that MLH1 methylation was patchy and/or monoallelic in region C of the MLH1 promoter by using combined bisulphite-restriction analysis (COBRA, Hitchins *et al*, 2007) (data not shown). This is consistent with the finding that MSI is extremely rare in NSCLC.

It is intriguing therefore, that *MLH1* methylation showed strong prognostic significance, which is reported here for the first time. It was a marker of poor survival in the whole cohort, and particularly in the LCC subgroup, with both univariate and bivariate analyses. This is in contrast to colorectal adenocarcinoma where *MLH1* methylation causes the MSI-H phenotype, which has improved prognosis. There was a high correlation between *DLEC1* and *MLH1* methylation (*P*=0.0002). As for *DLEC1*, *MLH1* methylation was associated with stage II tumours and spread to regional lymph nodes. Concordant methylation of *MLH1* and *DLEC1* was also a marker of poor prognosis independent of stage in the whole cohort (Table 4).

The close correlation between *MLH1* and *DLEC1* methylation may be a consequence or a byproduct of a long-range epigenetic effect in this region of chromosome 3p. The first such chromosomal region reported was 2q14.2, which shows modification of chromatin structure such as histone H3-K9 methylation in colon cancer cells. This results in clusters of both methylated and unmethylated genes being coordinately suppressed (Frigola *et al*, 2006). It has recently been shown that *DLEC1* and *MLH1* are also subject to long-range epigenetic regulation in colon cancer (Hitchins *et al*, 2007). Multiple genes in this region can be simultaneously silenced through promoter hypermethylation and histone methylation in MSI-positive colorectal cancers. This effect appears to extend centromeric from the *MLH1* gene and does not always reach *DLEC1* in all specimens. In bladder cancer there is also evidence of such long-range epigenetic regulation around the *DLEC1* gene, but here the predominant mechanism is gene silencing through histone methylation rather than CpG methylation, and *MLH1* was not analysed (Stransky *et al*, 2006). The two genes, which were analysed in both studies, *DLEC1* and its neighbour *PLCD1*, are silenced through DNA methylation and H3-K9 dimethylation in colorectal cancer whereas in bladder cancer they are silenced through histone H3-K9 trimethylation. This suggests that there are tissue-specific differences in this regulation. Therefore, if such long-range epigenetic regulation of chromosome 3p is also operating in lung cancer, it is possible that some genes in the region may be affected less than others. As a consequence the overall methylation in this region could serve as a marker of poorer prognosis but only some genes show complete loss of function.

The other three genes analysed in this study *RASSF1A*, *BLU* and *RARβ* are known to be methylated in lung cancer and all have shown functional characteristics of tumour suppressor genes (Toulouse *et al*, 2000; Shivakumar *et al*, 2002; Agathanggelou *et al*, 2003). *RARβ* is located 12 Mb telomeric from *MLH1*, and *RASSF1A* and *BLU* about 12 Mb centromeric from *DLEC1*. *RASSF1* methylation was also highly correlated with *DLEC1* (*P*=0.0003) and *MLH1* methylation (*P*=0.0006), whereas *RARβ* was methylated independent of the other genes. The correlation between *RASSF1A* and *BLU* methylation observed here (*P*=0.017) has also been described previously (Agathanggelou *et al*, 2003). However, none of these three markers were as strongly prognostic as *DLEC1* and *MLH1* methylation in this cohort. In a previous study *RASSF1A* methylation correlated with poor survival (Tomizawa *et al*, 2002), but this has not been confirmed in all cohorts (Toyooka *et al*, 2004; Choi *et al*, 2005). Here, *RASSF1A* methylation was a prognostic marker in univariate analyses but not independent of stage, as was also observed previously (Choi *et al*, 2005). It was interesting that concordant methylation of *MLH1* with *RASSF1* was an independent marker of poor prognosis. This suggests that a possible long-range epigenetic effect may extend centromeric from the *DLEC1* locus but not telomeric from the *MLH1* locus.

Taken together, our study has described two new prognostic markers, methylation of *DLEC1* and *MLH1* on chromosome 3p. Methylation of these two genes is clearly associated with each other and with methylation of *RASSF1* and *BLU*, which are ∼12 Mb centromeric from *DLEC1*. *MLH1* methylation itself does not lead to gene silencing in lung cancer and the biological significance of *DLEC1* methylation also needs further study. In any case, concordant methylation of *MLH1* with *DLEC1* or *RASSF1A* is a valuable prognostic indicator in lung cancer. Future studies should reveal whether *DLEC1*, another gene or perhaps multiple genes in this region are functionally the most important in lung carcinogenesis.

## Figures and Tables

**Table 1:** PCR primers used in the promoter methylation analysis of *RARβ*, *MLH1*, *DLEC1*, *RASSF1A* and *BLU*

**Gene**	**Forward primer**	**Reverse primer**
*RARβ-m* a	5′-TCGAGAACGCGAGCGATTC-3′	5′-GACCAATCCAACCGAAACGA-3′
*MLH1-m*	5′-AGCGATTTTTAACGCGTAAGC-3′	5′-CTCAATACCTCGTACTCACG-3′
*DLEC1-m*	5′-TTTCGTTGCGTATTTAAGATATTTC-3′	5′-CGTAACGCTCATTCTCGCTACC-3′
*DLEC1-u* b	5′-TAGTTTTGTAGTTTGGTTTTGTT-3′	5′-ACAAAATATCTTAAATACACAACA-3′
*RASSF1A-m*	5′-TTAGCGAAGTACGGGTTTAATC-3′	5′-CTACCGTATAAAATTACACGCG-3′
*BLU-m*	5′-CGTGGGTTATAGTTCGAGAAAGC-3′	5′-AACGAATTAACCGCGCCTACGC-3′
*MYOD1* c	5′-CCAACTCCAAATCCCCTCTCTAT-3′	5′-TGATTAATTTAGATTGGGTTTAGAGAAGGA-3′

a Primers specific for methylated, bisulphite converted DNA.

b Primers specific for unmethylated, bisulphite converted DNA.

c Control primers not including CpG sites specific for bisulphite converted DNA.

**Table 2:** Association of clinicopathologic variables with poor overall survival and with *RARβ*, *MLH1*, *DLEC1*, *RASSF1A* and *BLU* promoter methylation in the cohort of 239 NSCLC patients.

	**Kaplan– Meier log-rank**	**RAR*β* methylation**			**hMLH1 methylation**			**DLEC1 methylation**			**RASSF1A methylation**			**BLU methylation**		
		**No. of patients**			**No. of patients**			**No. of patients**			**No. of patients**			**No. of patients**		
**Clinicopathologic parameters**	***P*-value**	**+^a^**	**−b**	***P*-value**	+	−	***P*-value**	+	−	***P*-value**	+	−	***P*-value**	+	−	***P*-value**
*Age*	0.5754															
<68 (*n*=117)		63	54	0.4706	39	78	0.4032	49	68	0.3567	41	76	0.4371	34	83	0.0397
>68 (*n*=122)		60	62		47	75		44	78		37	85		51	71	
*Gender*	0.1680															
Male (*n*=155)		76	79	0.3068	57	98	0.7293	61	94	0.8488	49	106	0.6468	53	102	0.5475
Female (*n*=84)		47	37		29	55		32	52		29	55		32	52	
*Tumour differentiation*	0.2864															
Poor (*n*=103)		48	55	0.3388	44	59	0.3106	44	59	0.7712	35	68	0.3738	34	69	0.2847
Moderate (*n*=113)		65	48		35	78		41	72		37	76		41	72	
Well (*n*=20)		9	11		6	14		7	13		4	16		10	10	
Unknown (*n*=3)		1	2		1	2		1	2		2	1		0	3	
*Histologic type*	0.5207															
ADC (*n*=92)		56	36	0.0317	21	71	0.0047	29	63	0.0696	25	67	0.0008	42	50	0.0278
LCC (*n*=54)		20	34		22	32		19	35		29	25		17	37	
SCC (*n*=92)		47	45		42	50		44	48		23	69		25	67	
*Primary tumour stage*	0.1014															
T1 (*n*=64)		31	33	0.8517	16	48	0.0995	17	47	0.0555	19	45	0.8406	22	42	0.7303
T2 (*n*=154)		81	73		62	92		66	88		52	102		57	97	
T3 (*n*=21)		11	10		8	13		10	11		7	14		6	15	
*Regional lymph node status*	<0.0001															
N0 (*n*=174)		91	83	0.6728	54	120	0.0091	53	121	<0.0001	52	122	0.1378	68	106	0.0632
N1 (*n*=65)		32	33		32	33		40	25		26	39		17	48	
*AJCC staging*	<0.0001															
Stage I (*n*=153)		80	73	0.7341	46	107	0.0110	43	110	<0.0001	45	108	0.1562	62	91	0.0327
Stage II (*n*=86)		43	43		40	46		50	36		33	53		23	63	
																
Total		123	116		86	153		93	146		78	161		85	154	

a +Indicates number of patients with a methylated tumour.

b −Indicates number of patients with no methylation detected.

**Table 3:** *P*-values for pairwise correlation of promoter methylation in the 3p genes

	***MLH1***	***DLEC1***	***RASSF1A***	***BLU***
	***P*-value**	***P*-value**	***P*-value**	***P*-value**
*RARβ*	0.0691	0.1032	0.5543	0.7343
*MLH1*	—	0.0002	0.0006	0.0005
*DLEC1*	—	—	0.0003	0.0550
*RASSF1A*	—	—	—	0.0173

**Table 4:** Association of gene promoter methylation with survival in a univariate and a bivariate analysis, which takes into account AJCC stage of cancer

	**Cox Proportional Regression Model**							
	**Univariate**	**Bivariate**	**Univariate**	**Bivariate**	**Univariate**	**Bivariate**	**Univariate**	**Bivariate**
**Clinical or molecular marker**	**All samples (No.=239)**		**ADC (No.=92)**		**LCC (No.=54)**		**SCC (No.=92)**	
*AJCC stage*								
HR	2.795	2.580–2.728	3.126	2.767–3.042	2.930	2.438–3.256	2.594	2.411–2.597
95% CI	2.009–3.889		1.808–5.404		1.417–6.056		1.506–4.467	
* P*	<0.0001	<0.0001	<0.0001	<0.0007	0.004	<0.021	0.0006	<0.002
								
*MLH1/DLEC1*								
HR	2.075	1.668	2.347	1.415	2.146	2.284	1.731	1.525
95% CI	1.428–3.015	1.138–2.447	1.210–4.555	0.683–2.932	0.939–4.906	0.992–5.257	0.984–3.043	0.863–2.696
*P*	0.0001	0.009	0.012	0.350	0.070	0.052	0.057	0.146
								
*MLH1/RASSF1A*								
HR	1.951	1.688	1.702	1.245	3.011	3.223	1.390	1.206
95% CI	1.307–2.913	1.127–2.529	0.832–3.483	0.595–2.607	1.395–6.501	1.482–7.008	0.677–2.854	0.586–2.482
*P*	0.001	0.011	0.146	0.561	0.005	0.003	0.370	0.611
								
*DLEC1/RASSF1A*								
HR	1.505	1.137	1.763	1.102	1.292	0.925	1.338	1.213
95% CI	1.015–2.233	0.759–1.705	0.927–3.352	0.548–2.214	0.574–2.909	0.395–2.163	0.686–2.607	0.623–2.362
*P*	0.042	0.533	0.084	0.785	0.536	0.857	0.393	0.571
								
*MLH1*								
HR	1.621	1.421	2.034	1.512	2.591	2.926	1.007	0.972
95% CI	1.164–2.257	1.016–1.988	1.171–3.535	0.842–2.714	1.220–5.499	1.358–6.308	0.597–1.697	0.576–1.640
*P*	0.004	0.040	0.012	0.166	0.013	0.006	0.980	0.915
								
*DLEC1*								
HR	1.783	1.471	1.681	1.337	1.454	1.103	1.968	1.754
95% CI	1.283–2.479	1.050–2.062	0.983–2.872	0.766–2.333	0.698–3.029	0.514–2.366	1.155–3.353	1.023–3.007
*P*	0.0006	0.025	0.058	0.306	0.317	0.802	0.013	0.041
								
*RASSF1A*								
HR	1.474	1.259	1.397	1.174	2.453	1.902	1.138	1.021
95% CI	1.049–2.071	0.892–1.776	0.796–2.451	0.661–2.084	1.133–5.310	0.856–4.230	0.620–2.089	0.556–1.876
*P*	0.025	0.190	0.244	0.584	0.023	0.115	0.676	0.947

Abbreviations: AJCC=American Joint Committee on Cancer; HR=Hazard Ratio; CI=confidence interval.

## References

[bib1] Agathanggelou A, Dallol A, Zochbauer-Muller S, Morrissey C, Honorio S, Hesson L, Martinsson T, Fong KM, Kuo MJ, Yuen PW, Maher ER, Minna JD, Latif F (2003) Epigenetic inactivation of the candidate 3p21.3 suppressor gene BLU in human cancers. Oncogene 22: 1580–1588 12629521 10.1038/sj.onc.1206243

[bib2] Belinsky SA, Palmisano WA, Gilliland FD, Crooks LA, Divine KK, Winters SA, Grimes MJ, Harms HJ, Tellez CS, Smith TM, Moots PP, Lechner JF, Stidley CA, Crowell RE (2002) Aberrant promoter methylation in bronchial epithelium and sputum from current and former smokers. Cancer Res 62: 2370–2377 11956099

[bib3] Benachenhou N, Guiral S, Gorska-Flipot I, Labuda D, Sinnett D (1998) High resolution deletion mapping reveals frequent allelic losses at the DNA mismatch repair loci hMLH1 and hMSH3 in non-small cell lung cancer. Int J Cancer 77: 173–180 9650548 10.1002/(sici)1097-0215(19980717)77:2<173::aid-ijc1>3.0.co;2-n

[bib4] Choi N, Son DS, Song I, Lee HS, Lim YS, Song MS, Lim DS, Lee J, Kim H, Kim J (2005) RASSF1A is not appropriate as an early detection marker or a prognostic marker for non-small cell lung cancer. Int J Cancer 115: 575–581 15700308 10.1002/ijc.20916

[bib5] Cooper WA, Kohonen-Corish MRJ, Chan C, Kwun SY, McCaughan B, Kennedy C, Sutherland RL, Lee C-S (2008) Prognostic significance of DNA repair proteins MLH1, MSH2 and MGMT expression in non-small cell lung cancer and precursor lesions. Histopathology 52: 613–622 18370958 10.1111/j.1365-2559.2008.02999.x PMC2325921

[bib6] Daigo Y, Nishiwaki T, Kawasoe T, Tamari M, Tsuchiya E, Nakamura Y (1999) Molecular cloning of a candidate tumor suppressor gene, DLC1, from chromosome 3p21.3. Cancer Res 59: 1966–1972 10213508

[bib7] Dammann R, Li C, Yoon JH, Chin PL, Bates S, Pfeifer GP (2000) Epigenetic inactivation of a RAS association domain family protein from the lung tumour suppressor locus 3p21.3. Nature Genet 25: 315–319 10888881 10.1038/77083

[bib8] Eads CA, Danenberg KD, Kawakami K, Saltz LB, Danenberg PV, Laird PW (1999) CpG island hypermethylation in human colorectal tumors is not associated with DNA methyltransferase overexpression. Cancer Res 59: 2302–2306 10344733

[bib9] Esteller M, Sanchez-Cespedes M, Rosell R, Sidransky D, Baylin SB, Herman JG (1999) Detection of aberrant promoter hypermethylation of tumor suppressor genes in serum DNA from non-small cell lung cancer patients. Cancer Res 59: 67–70 9892187

[bib10] Frigola J, Song J, Stirzaker C, Hinshelwood RA, Peinado MA, Clark SJ (2006) Epigenetic remodeling in colorectal cancer results in coordinate gene suppression across an entire chromosome band. Nature Genet 38: 540–549 16642018 10.1038/ng1781

[bib11] Grondin SC, Liptay MJ (2002) Current concepts in the staging of non-small cell lung cancer. Surg Oncol 11: 181–190 12450554 10.1016/s0960-7404(02)00050-6

[bib12] Hitchins MP, Lin VA, Buckle A, Cheong K, Halani N, Ku S, Kwok CT, Packham D, Suter CM, Meagher A, Stirzaker C, Clark S, Hawkins NJ, Ward RL (2007) Epigenetic inactivation of a cluster of genes flanking MLH1 in microsatellite-unstable colorectal cancer. Cancer Res 67: 9107–9116 17909015 10.1158/0008-5472.CAN-07-0869

[bib13] Hsu HS, Wen CK, Tang YA, Lin RK, Li WY, Hsu WH, Wang YC (2005) Promoter hypermethylation is the predominant mechanism in hMLH1 and hMSH2 deregulation and is a poor prognostic factor in nonsmoking lung cancer. Clin Cancer Res 11: 5410–5416 16061855 10.1158/1078-0432.CCR-05-0601

[bib14] Hung J, Kishimoto Y, Sugio K, Virmani A, McIntire DD, Minna JD, Gazdar AF (1995) Allele-specific chromosome 3p deletions occur at an early stage in the pathogenesis of lung carcinoma. JAMA 273: 558–563 7837389

[bib15] Ishikawa S, Kai M, Tamari M, Takei Y, Takeuchi K, Bandou H, Yamane Y, Ogawa M, Nakamura Y (1997) Sequence analysis of a 685-kb genomic region on chromosome 3p22–p21.3 that is homozygously deleted in a lung carcinoma cell line. DNA Res 4: 35–43 9179494 10.1093/dnares/4.1.35

[bib16] Ito M, Ito G, Kondo M, Uchiyama M, Fukui T, Mori S, Yoshioka H, Ueda Y, Shimokata K, Sekido Y (2005) Frequent inactivation of RASSF1A, BLU, and SEMA3B on 3p21.3 by promoter hypermethylation and allele loss in non-small cell lung cancer. Cancer Lett 225: 131–139 15922865 10.1016/j.canlet.2004.10.041

[bib17] Kohonen-Corish MR, Cooper WA, Saab J, Thompson JF, Trent RJ, Millward MJ (2006) Promoter hypermethylation of the O(6)-methylguanine DNA methyltransferase gene and microsatellite instability in metastatic melanoma. J Inv Dermatol 126: 167–171 10.1038/sj.jid.5700005 16417233

[bib18] Kohonen-Corish MR, Daniel JJ, Chan C, Lin BP, Kwun SY, Dent OF, Dhillon VS, Trent RJ, Chapuis PH, Bokey EL (2005) Low microsatellite instability is associated with poor prognosis in stage C colon cancer. J Clin Oncol 23: 2318–2324 15800322 10.1200/JCO.2005.00.109

[bib19] Kohonen-Corish MR, Sigglekow ND, Susanto J, Chapuis PH, Bokey EL, Dent OF, Chan C, Lin BP, Seng TJ, Laird PW, Young J, Leggett BA, Jass JR, Sutherland RL (2007) Promoter methylation of the mutated in colorectal cancer gene is a frequent early event in colorectal cancer. Oncogene 26: 4435–4441 17260021 10.1038/sj.onc.1210210

[bib20] Kwong J, Chow LS, Wong AY, Hung WK, Chung GT, To KF, Chan FL, Daigo Y, Nakamura Y, Huang DP, Lo KW (2007) Epigenetic inactivation of the deleted in lung and esophageal cancer 1 gene in nasopharyngeal carcinoma. Genes Chrom Cancer 46: 171–180 17099870 10.1002/gcc.20398

[bib21] Kwong J, Lee JY, Wong KK, Zhou X, Wong DT, Lo KW, Welch WR, Berkowitz RS, Mok SC (2006) Candidate tumor-suppressor gene DLEC1 is frequently downregulated by promoter hypermethylation and histone hypoacetylation in human epithelial ovarian cancer. Neoplasia 8: 268–278 16756719 10.1593/neo.05502 PMC1600675

[bib22] Millar DS, Warnecke PM, Melki JR, Clark SJ (2002) Methylation sequencing from limiting DNA: embryonic, fixed, and microdissected cells. Methods 27: 108–113 12095267 10.1016/s1046-2023(02)00061-0

[bib23] Okuda T, Kawakami K, Ishiguro K, Oda M, Omura K, Watanabe G (2005) The profile of hMLH1 methylation and microsatellite instability in colorectal and non-small cell lung cancer. Int J Mol Med 15: 85–90 15583832

[bib24] Palmisano WA, Divine KK, Saccomanno G, Gilliland FD, Baylin SB, Herman JG, Belinsky SA (2000) Predicting lung cancer by detecting aberrant promoter methylation in sputum. Cancer Res 60: 5954–5958 11085511

[bib25] Safar AM, Spencer III H, Su X, Coffey M, Cooney CA, Ratnasinghe LD, Hutchins LF, Fan CY (2005) Methylation profiling of archived non-small cell lung cancer: a promising prognostic system. Clin Cancer Res 11: 4400–4405 15958624 10.1158/1078-0432.CCR-04-2378

[bib26] Shivakumar L, Minna J, Sakamaki T, Pestell R, White MA (2002) The RASSF1A tumor suppressor blocks cell cycle progression and inhibits cyclin D1 accumulation. Mol Cell Biol 22: 4309–4318 12024041 10.1128/MCB.22.12.4309-4318.2002 PMC133879

[bib27] Sinicrope FA, Rego RL, Halling KC, Foster N, Sargent DJ, La Plant B, French AJ, Laurie JA, Goldberg RM, Thibodeau SN, Witzig TE (2006) Prognostic impact of microsatellite instability and DNA ploidy in human colon carcinoma patients. Gastroenterology 131: 729–737 16952542 10.1053/j.gastro.2006.06.005

[bib28] Stransky N, Vallot C, Reyal F, Bernard-Pierrot I, de Medina SG, Segraves R, de Rycke Y, Elvin P, Cassidy A, Spraggon C, Graham A, Southgate J, Asselain B, Allory Y, Abbou CC, Albertson DG, Thiery JP, Chopin DK, Pinkel D, Radvanyi F (2006) Regional copy number-independent deregulation of transcription in cancer. Nature Genet 38: 1386–1396 17099711 10.1038/ng1923

[bib29] Suraweera N, Duval A, Reperant M, Vaury C, Furlan D, Leroy K, Seruca R, Iacopetta B, Hamelin R (2002) Evaluation of tumor microsatellite instability using five quasimonomorphic mononucleotide repeats and pentaplex PCR. Gastroenterology 123: 1804–1811 12454837 10.1053/gast.2002.37070

[bib30] Tomizawa Y, Kohno T, Kondo H, Otsuka A, Nishioka M, Niki T, Yamada T, Maeshima A, Yoshimura K, Saito R, Minna JD, Yokota J (2002) Clinicopathological significance of epigenetic inactivation of RASSF1A at 3p21.3 in stage I lung adenocarcinoma. Clin Cancer Res 8: 2362–2368 12114441

[bib31] Toulouse A, Morin J, Dion PA, Houle B, Bradley WE (2000) RARbeta2 specificity in mediating RA inhibition of growth of lung cancer-derived cells. Lung Cancer 28: 127–137 10717330 10.1016/s0169-5002(99)00122-1

[bib32] Toyooka S, Suzuki M, Maruyama R, Toyooka KO, Tsukuda K, Fukuyama Y, Iizasa T, Aoe M, Date H, Fujisawa T, Shimizu N, Gazdar AF (2004) The relationship between aberrant methylation and survival in non-small cell lung cancers. Br J Cancer 91: 771–774 15266335 10.1038/sj.bjc.6602013 PMC2364802

[bib33] Usadel H, Brabender J, Danenberg KD, Jeronimo C, Harden S, Engles J, Danenberg PV, Yang S, Sidransky D (2002) Quantitative adenomatous polyposis coli promoter methylation analysis in tumor tissue, serum, and plasma DNA of patients with lung cancer. Cancer Res 62: 371–375 11809682

[bib34] Virmani AK, Rathi A, Zochbauer-Muller S, Sacchi N, Fukuyama Y, Bryant D, Maitra A, Heda S, Fong KM, Thunnissen F, Minna JD, Gazdar AF (2000) Promoter methylation and silencing of the retinoic acid receptor-beta gene in lung carcinomas. J Natl Cancer Inst 92: 1303–1307 10944551 10.1093/jnci/92.16.1303

[bib35] Weisenberger DJ, Siegmund KD, Campan M, Young J, Long TI, Faasse MA, Kang GH, Widschwendter M, Weener D, Buchanan D, Koh H, Simms L, Barker M, Leggett B, Levine J, Kim M, French AJ, Thibodeau SN, Jass J, Haile R, Laird PW (2006) CpG island methylator phenotype underlies sporadic microsatellite instability and is tightly associated with BRAF mutation in colorectal cancer. Nature Genet 38: 787–793 16804544 10.1038/ng1834

[bib36] Wistuba II, Behrens C, Virmani AK, Mele G, Milchgrub S, Girard L, Fondon III JW, Garner HR, McKay B, Latif F, Lerman MI, Lam S, Gazdar AF, Minna JD (2000) High resolution chromosome 3p allelotyping of human lung cancer and preneoplastic/preinvasive bronchial epithelium reveals multiple, discontinuous sites of 3p allele loss and three regions of frequent breakpoints. Cancer Res 60: 1949–1960 10766185

[bib37] Xinarianos G, Liloglou T, Prime W, Maloney P, Callaghan J, Fielding P, Gosney JR, Field JK (2000) hMLH1 and hMSH2 expression correlates with allelic imbalance on chromosome 3p in non-small cell lung carcinomas. Cancer Res 60: 4216–4221 10945633

[bib38] Yanagawa N, Tamura G, Oizumi H, Takahashi N, Shimazaki Y, Motoyama T (2003) Promoter hypermethylation of tumor suppressor and tumor-related genes in non-small cell lung cancers. Cancer Sci 94: 589–592 12841866 10.1111/j.1349-7006.2003.tb01487.x PMC11160194

[bib39] Zabarovsky ER, Lerman MI, Minna JD (2002) Tumor suppressor genes on chromosome 3p involved in the pathogenesis of lung and other cancers. Oncogene 21: 6915–6935 12362274 10.1038/sj.onc.1205835

[bib40] Zochbauer-Muller S, Fong KM, Maitra A, Lam S, Geradts J, Ashfaq R, Virmani AK, Milchgrub S, Gazdar AF, Minna JD (2001) 5′ CpG island methylation of the FHIT gene is correlated with loss of gene expression in lung and breast cancer. Cancer Res 61: 3581–3585 11325823

